# Supplementary figures and images for: Blood vessel occlusion by Cryptococcus neoformans is a mechanism for haemorrhagic dissemination of infection
Source: PLoS Pathog. 2022 Apr 21;18(4):e1010389. doi: 10.1371/journal.ppat.1010389 (PMC9022829; doi:10.1371/journal.ppat.1010389)

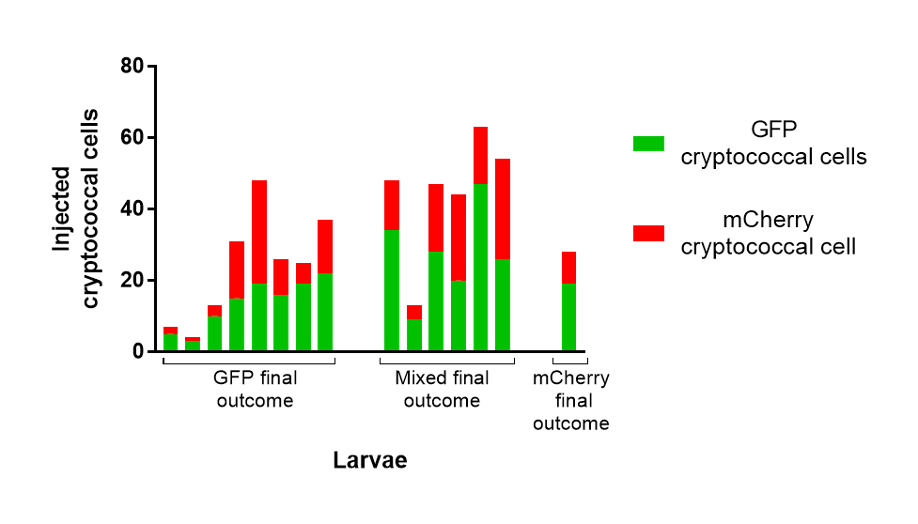

Supplement: S1 Fig — Infection of AB wild-type larvae with 5:1 ratio of GFP:mCherry KN99 C. neoformans, actual number of cryptococcal cells, both GFP and mCherry KN99 in 25 cfu injected grouped by majority colour outcome. Each bar represents an individual fish. (TIF) [file ppat.1010389.s001.tif]

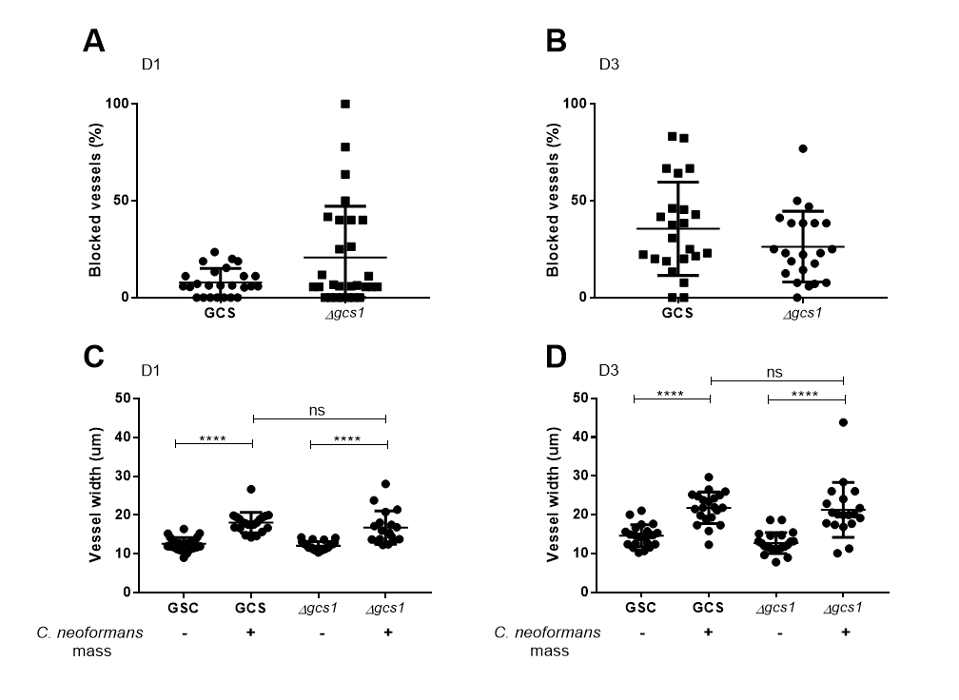

Supplement: S2 Fig — A-D: Infection of KDRL mCherry blood marker transgenic line with 1000 cfu Δgsc or its parental strain C. neoformans A Blocked vessels (% of all inter-segmental vessels) at 1 dpi (n = 2, +/- SD, Kruskal-Wallis test) B Blocked vessels (% of all inter-segmental vessels) at 3 dpi (n = 2, +/- SD, Kruskal-Wallis test) C Vessel width with or without C. neoformans at 1 dpi (n = 2, +/- SD, ns = not significant, ****p<0.0001, Kruskal-Wallis test) D Vessel width with or without C. neoformans at 3 dpi (n = 2, +/- SD, ns = not significant, ****p<0.0001, Kruskal-Wallis test) (TIF) [file ppat.1010389.s002.tif]

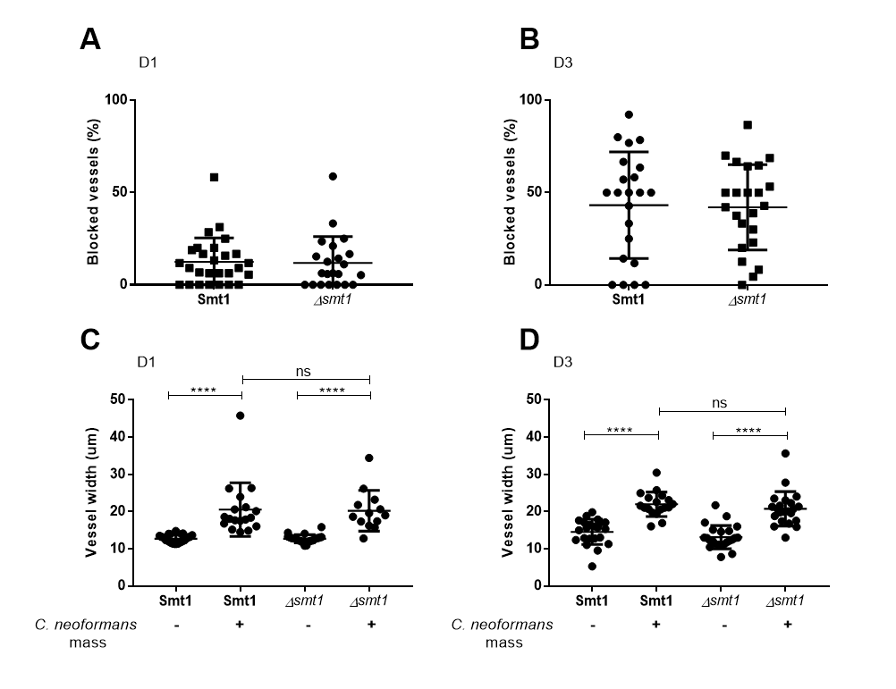

Supplement: S3 Fig — A-D: Infection of KDRL mCherry blood marker transgenic line with 1000 cfu Δsmt or its parental strain C. neoformans A Blocked vessels (% of all inter-segmental vessels) at 1 dpi (n = 2, +/- SD, Kruskal-Wallis test) B Blocked vessels (% of all inter-segmental vessels) at 3 dpi (n = 2, +/- SD, Kruskal-Wallis test) C Vessel width with or without C. neoformans at 1 dpi (n = 2, +/- SD, ns = not significant, ****p<0.0001, Kruskal-Wallis test) D Vessel width with or without C. neoformans at 3 dpi (n = 2, +/- SD, ns = not significant, ****p<0.0001, Kruskal-Wallis test) (TIF) [file ppat.1010389.s003.tif]

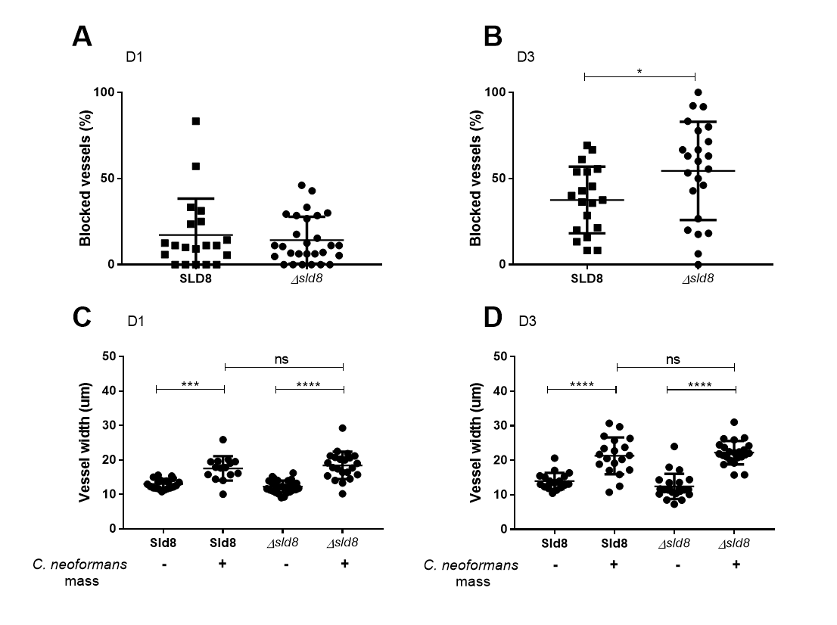

Supplement: S4 Fig — A-D: Infection of KDRL mCherry blood marker transgenic line with 1000 cfu Δsld8 or its parental strain C. neoformans A Blocked vessels (% of all inter-segmental vessels) at 1 dpi (n = 2, +/- SD, Kruskal-Wallis test) B Blocked vessels (% of all inter-segmental vessels) at 3 dpi (n = 2, +/- SD, *p<0.05, Kruskal-Wallis test) C Vessel width with or without C. neoformans at 1 dpi (n = 2, +/- SD, ns = not significant, ****p<0.0001, Kruskal-Wallis test) D Vessel width with or without C. neoformans at 3 dpi (n = 2, +/- SD, ns = not significant, ****p<0.0001, Kruskal-Wallis test) (TIF) [file ppat.1010389.s004.tif]

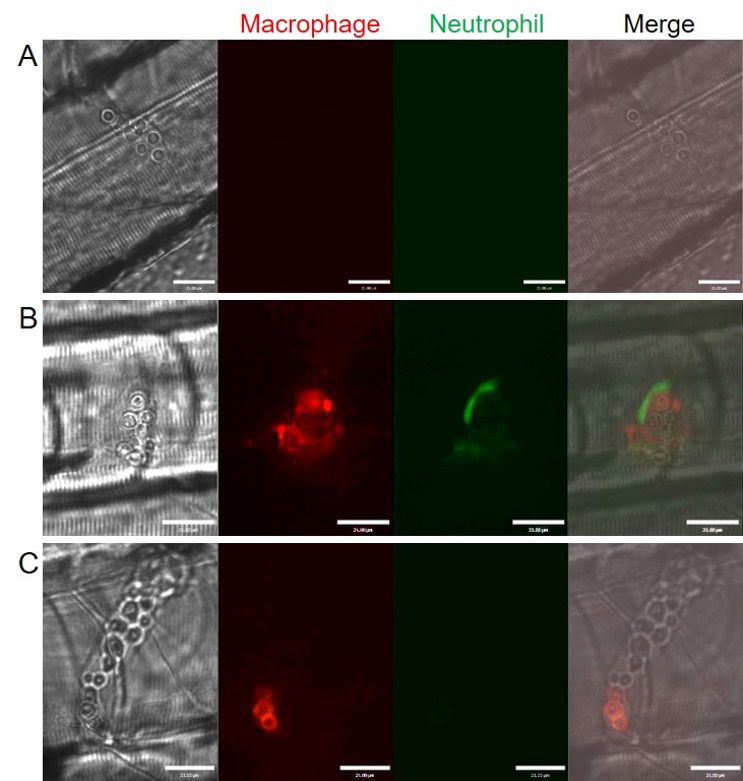

Supplement: S5 Fig — A-C: Infection of Tg(mpeg1:mCherry.CAAX)sh378 stably crossed to Tg(mpx:eGFP)i114 larvae at 2 dpf with 1000 cfu KN99 C. neoformans imaged at 3 dpi A Example image of cryptococcal mass within inter-segmental vessel of larvae with no phagocyte recruitment B Example image of cryptococcal mass within inter-segmental vessel of larvae with both macrophage and neutrophil recruitment C Example image of cryptococcal mass within inter-segmental vessel of larvae with macrophage recruitment, also showing typical phagocytosis of just part of the cryptococcal mass. (TIF) [file ppat.1010389.s005.tif]
