# Supplementary material for: Blood vessel occlusion by Cryptococcus neoformans is a mechanism for haemorrhagic dissemination of infection
Source: PLoS Pathog. 2022 Apr 21;18(4):e1010389. doi: 10.1371/journal.ppat.1010389 (PMC9022829; doi:10.1371/journal.ppat.1010389)
Supplement: Attachment — Submitted filename: Response to reviewers.pdf [file ppat.1010389.s008.pdf]

Response to Reviewers

Part I – Summary:

Reviewer #1: Gibson and Bojarczuk et al present a very interesting manuscript describing how intravascular growth of Cryptococcus neoformans (Cn) correlates with the appearance of endothelial damage and preceding Cn invasion out of the vasculature. They make excellent use of dual fluorescent Cn to correlate individual instances of intravascular growth with localised pathologies. The second half of the manuscript focusses nicely on the Cn-endothelial interaction and drills down into VE-cadherin junctional stiffness as a mechanistic determinant of tissue invasion.

Reviewer #2: The authors investigated the mechanical basis of C. neoformans dissemination to the brain using a zebrafish model. The findings are interesting, but their biological relevance of their model remains unclear.

Reviewer #3: This manuscript by Gibson et al. describes an exciting new finding that the physical occlusion of vasculature by cryptococcomas leads to enhanced vascular tension and rupture. These rupture events lead to local dissemination of Cryptococcus within the animal. Additionally, they demonstrate that enhanced vascular stiffness can increase dissemination of Cryptococcus. These findings offer an interesting new mechanism by which Cryptococcus may drive its dissemination. These findings are also of broad interest to researchers focused on other infectious diseases because they illustrate a new mechanistic approach to dissemination and, as the authors note, there are signs that other pathogens may also drive similar vascular rupture. While the larger ideas of the paper are compelling, there are substantial gaps in the data to support such a conclusion. In particular, the authors need substantial new data to definitively show that it is rupture that is leading to release of cryptococci and not either of the other two mechanisms of dissemination - the Trojan horse mechanism or transcytosis that are mediating this with subsequent secondary vascular rupture.

> **Response:** We thank all the reviewers for their interest in our new cryptococcal dissemination mechanism data. In addition, we thank the reviewers for their thoughtful and very useful feedback, which we address in detail below. We believe our manuscript has benefitted greatly from our response to the reviewers' comments, specifically with regard to further elucidation of the mechanistic dissemination process in relation to changes in blood flow and ruling out a role for Trojan horses, as well as clarification of how our proposed mechanism is relevant for human cryptococcal disease.

Part II – Major Issues: Key Experiments Required for Acceptance

Reviewer #1:

1. It is not clear how the analysis in Fig 3C supports the interpretation that cryptococcal expansion occurs in the small vessels. The proposed interpretation would require correlation of an overwhelming GFP outcome with animals that have 100% GFP small vessel regardless (but preferably despite) of having mCherry in the large vessels.
Fig 3D attempts to perform this correlation but it is not clear from the text if only initial small vessel cryptococcal masses are analysed or if initial intravascular masses from any vessel type are used. I still feel that this question is better answered by either sequential or

continuous live imaging which the authors have previously used with great effect in establishing the zebrafish-Cn platform.

Response: We thank reviewer 1 for these useful comments.

In Fig. 3C we are demonstrating that the ratio of GFP:mCherry cryptococcal cells is skewed primarily to a single cryptococcal colour (GFP) in narrow blood vessels, supporting our conclusion that clonal expansion occurs in these vessels; if clonal expansion was not occurring within these narrow vessels, we would expect to have only mixed outcomes, as we see for the large caudal vein. This, together with data from Fig 1, supports the suggestion that clonal expansion occurs in narrow blood vessels after a cryptococcal trapping event. In order to show this more clearly, we have added another method of analysis (new Fig. 3D) which shows the percentage of fish with single or mixed coloured strains in larger or narrow vessels, clearly showing narrow blood vessels are much more likely to be a single colour in comparison to large vessels (p=<0.0001).

Fig. 3D (now Fig. 3E) already presented an analysis of masses that were observed only in inter-segmental vessels, and it has now been adjusted to state this explicitly in the legend.

With regard to sequential imaging, Fig. 1C demonstrates the growth of cryptococcal masses within intersegmental vessels over time using the same method we have previously published to which the reviewer is referring. It is not feasible to do continuous imaging over the whole experiment with live zebrafish because of the growth of larvae, intolerance of continuous anaesthetic and very large data files (hundreds of TB). However, to further support our conclusion we have added a new video (Video 1, line 181), which clearly demonstrates individual cryptococcal budding events at a cryptococcal mass site within an intersegmental vessel.

2. Assessment of vascular integrity in Fig 6C 6E: Dextran permeability assay is a better measure of vascular barrier function than the KDRL:mCherry transgenic.

Response: We agree that dextran permeability is a useful assay to support Fig. 6C and 6E, which indicate vessel damage. We have completed several imaging experiments with injections of dextran into zebrafish larvae at 5dpf (following CN infection at 2dpf), with limited success in seeing dextran leaks at sites of vessel blockages. We conclude that this is due to reduced blood flow within blocked vessels. A new image (Fig. 6F, line 299) has been included that shows intersegmental vessels blocked with a CN masses and one not blocked; dextran is clearly visible in the non-blocked vessel. In the blocked vessels, little if any dextran is seen. In many cases, we suggest that this has led to our not observing dextran leakage at mass sites. However, we have observed small amounts of dextran leakage at sites of extended cryptococcal somite dissemination (Fig. 6G and sentence, line 300) which suggests that major vessel damage has occurred at these locations allowing any small amount of dextran (present in blocked vessels) to spread into sites surrounding the cryptococcal mass. These new dextran assay data have been added to Figure 6 in the main body of the paper; however, we would be agreeable to move them into Supplemental data if considered more appropriate.

3. ADAM10 inhibitor in Figure 7D. Need to demonstrate efficacy of GI254023X on vascular phenotypes with VE-cadherin-TS transgenic embryos +/- infection.

Response: The vascular phenotype of GI254023X has been demonstrated previously in zebrafish (Lo Sardo *et al.*, 2012) (reference also added line 337). Unfortunately, we were unable to maintain this complex transgenic during the Covid-19 pandemic related shutdown in the UK. Therefore, while we agree with the reviewer that their proposed experiment would be interesting, we think that the existing evidence is sufficient to support our conclusions.

Reviewer #2: The authors investigated the mechanical basis of C. neoformans dissemination to the brain using a zebrafish model. The findings are interesting, but their biological relevance of their model remains unclear.

a) For example, the findings were based on size and tension of blood vessels, but there was no mention of blood flow.
b) Also it remains unclear whether the proposed concept is relevant to the pathogenesis of C. neoformans CNS infection.
c) For example, the plb1 was shown to contribute to C. neoformans CNS infection in the mouse model, but did not increase the size of cryptococcal masses. In addition, the mutants, plb1 and lac1, showed increased blocked vessels at 1 dpi, but not at 3 dpi. Also, there was no citation on contribution of lac1 to C. neoformans CNS infection. These findings raised the question on whether the proposed concept is relevant to C. neoformans CNS infection.
d) Many discussions on infarcts and vasculitis in bacterial meningitis are not directly relevant to the authors' speculation of C. neoformans dissemination to the brain. In addition, this manuscript suffers from additional conceptual and technical issues as summarized below.

Response:

We thank the reviewer for these detailed comments.

a) The reviewer raises an important concern about our reduced blood flow conclusion. In the Results section, we had only imaged non-labelled blood cells becoming trapped behind cryptococci (Fig. 1D), which had suggested to us that these cryptococcal mass events inhibit blood flow. In order to support this further, we have now used a transgenic zebrafish line with fluorescent blood cells, to further visualise blood cell trapping at cryptococcal mass sites (new Fig 1E). Furthermore, the impact of blood flow is now mentioned at this location (line 116), as well as added in where the new dextran data shows blood flow disruption (line 296). We believe these data in total provide very good evidence for our conclusion that blood flow is disrupted at sites of cryptococcal masses.
b) We have amended the manuscript to increase the clarity of the relevance of the vessel damage and dissemination mechanism for human CNS infections. We have cited human patient data (paragraph starting line 70) in the introduction, as well as including brain vessel data within the zebrafish model (line 224, Fig 4H) showing our proposed mechanism can occur in the brains of larvae. In addition, we have now made this connection explicit in the discussion (line 351).
c) We apologise for the lack of clarity on the use of plb1 and lac1 cryptococcal mutants. For the plb1 data, we show that plb1 mutant cryptococcal masses are significantly larger than wild-type cryptococcal cells at 1 dpi (Fig. 5D), suggesting increased trapping events are based on the size phenotype we describe. Our data therefore do not contradict the mouse model data showing plb1 contributes to CNS dissemination. Inhalation models do not represent our blood infection model, especially since plb has been shown to be required for escape from lungs,

but not for dissemination from the blood into the brain (Santangelo *et al.*, 2004). Furthermore, strains expressing reduced plb remain able to disseminate from the blood into the brain in mice (Chen *et al.*, 1997). We have adjusted sentences (lines 244 and lines 250) for improved clarity on the roles of plb and lac in CNS dissemination and have also added citations on the role of lac1. With regard to direct relevance to cryptococcal infection, we include citations on how meningitis and infarcts are observed in human infections, for which we can now provide potential mechanism.

d) We agree that our data only addresses cryptococcal infections. However, we have chosen to include the discussions on infarcts and vasculitis in bacterial infections to provide possible broader implications of our results due to similarities in symptoms and infections observed in case reports. This reflects our interpretation of the function of the discussion section. However, if there is agreement between the reviewers and the journal editors, we would be willing to remove these elements from our discussion.

Minor issues:

1. line 108 (for Fig 1), similar sizes in vessels alone are not sufficient for justifying similar functions. It is known that biology of blood vessels differs based on the location, size and other parameters such as blood flow.

Response: We agree and have amended our sentence (line 107) for clarity to make it clear we now state "*suggesting a vessel size similar to cryptococcal cell size may play a role in trapping*"

2. line 135 (for Fig 2), it is unclear why mixed populations were used and whether there are any differences between GFP vs mCherry Cryptococci for their behaviour.

Response: We have added a sentence for clarity on the use of GFP and mCherry strains (line 136) and the reason for using mixed populations (to identify clonal populations) and the reason we used a 1:5, rather than 1:1, combination is explained (lines 138). We have added a sentence and citation, which demonstrate that the two fluorescent lines have corresponding growth and infection dynamics (line 137). We have also rewritten this entire section significantly for improved clarity.

3. line 151, it is unclear what is the biological meaning of clonal expansion of cryptococcal masses and skewing in blood vessel biology.

Response: We apologise for the lack of clarity. We have now amended the sentence (now line 132) to be clear that it refers to cryptococcal infection dynamics not to blood vessel biology.

4. line 247 (for Fig 6), description on vessel rupture and dissemination also needs biological explanation.

Response: The sentence has been adjusted for clarity (line 279) and citations added to provide further evidence.

5. line 259 (for Fig 6), it is unclear why macrophages count not be identified, perhaps need transgenic macrophage marker

Response: We thank the reviewer for this suggestion. We did not use a transgenic zebrafish line to visualise macrophage Trojan horse examples, as this dissemination mechanism has already been published. However, we have added data using clodronate treatment, showing that macrophages are not required for the dissemination events we describe (Fig 6K). We then used a dual macrophage and neutrophil transgenic line to show that the majority of cryptococcal masses are not associated with phagocytes (Fig 6L and M).

6. line 280. ADAM10 is pleiotropic and its chemical inhibition may not be specific for VE-cadherin.

Response: We thank the reviewer for this comment. We agree our data are suggestive rather than exhaustive and have adjusted line 335 wording to ensure we are clear that there may be off-target effects. In addition, previous work in zebrafish has shown this effect and this reference (Lo Sardo *et al.*, 2012) has been added (line 337).

7. Several figures are difficult to discern.

Response: We would be happy to revise figures at the editor's request but believe this is a problem with the processing following upload not the figure files themselves.

Reviewer #3: The main focus of this paper is the idea that cryptococcoma formation and enhanced vascular tension can drive dissemination either with or without vascular rupture. While this is a compelling idea and, consistent with this, the authors see dissemination at sites of cryptococcoma formation, the authors do not directly prove that rupture is the mechanism. As the authors note, there are several other routes by which Cryptococcus can cross vasculature - namely transcytosis and a Trojan horse mechanism using macrophages to carry them across. The authors see evidence for both of these mechanisms. The authors need to show that their new mechanism of dissemination occurs outside of these other dissemination mechanisms. The authors should perform longitudinal imaging in animals with labelled leukocyte and vascular populations at high temporal resolution to show that this vascular rupture is indeed a distinct mechanism and that they are not observing Cryptococcus disseminated through the macrophage/endothelial transcytosis routes and subsequent vasculature rupture.

Response: We thank reviewer 3 for these useful suggestions. We agree that it would be ideal to show vessel damage mediated dissemination occurs in the absence of other routes of cryptococcal dissemination (transcytosis or Trojan horse). However, it is extremely difficult to discount a role of transcytosis because high resolution imaging cannot be maintained in live animals for the time periods required (days) and the described dissemination events occur at a low frequency (as we described above for the response to Reviewer 1). In fact, we are the only research group that has been able to directly image at the required temporal and spatial resolution to measure the outcome of host pathogen interactions of cryptococcus during infection (e.g. Bojarczuk et al., 2016, Gilbert et al., 2017) It is therefore very unlikely we would have observed dissemination events occurring by this process. However, since we identify dissemination events where vessels are intact or damaged, this suggests that both transcytosis and damage mediated dissemination occurs. We also note in the

manuscript that trapping and expansion events likely promote transcytosis as well as causing vessel damage, as we suspect trapping may be linked to promoting transcytosis, as we mention in the results section (line 360).

To address the impact of Trojan horse dissemination, cryptococcal dissemination events in larvae, in the absence of macrophages (depleted with clodronate) (Fig. 6 K, line 296) show that dissemination can occur without macrophages present, with the observed increase in dissemination likely due to reduced infection control. This suggests these dissemination events do not require macrophages.

Response: We agree. Specifically, in order to support our image data suggesting vessel damage at sites of cryptococcal mass growth, we used both vessel tension (FRET transgenic lines) and chemical analysis (ADAM10 inhibitor) to examine vessel tension at sites of masses and how increased tension impacts upon dissemination. Furthermore, as above (reviewer #1, comment 2), we have used dextran to further analyse the vessel integrity (with some difficulty likely caused by reduced blood flow) but have added data showing some leakages at sites of dissemination and text to address this.

Similar to the previous concern, are any of the Cryptococcus in cryptococcomas inside of host immune cells or is the cryptococcoma an entirely extracellular structure? While the authors refer to them as Cryptococcus masses and show that the larger cluster is surrounded by capsule, suggesting that they are most likely extracellular, it is important for the authors to show whether they localize into macrophage/neutrophil populations, as the finding that these Cryptococcus populations are within host immune cells would fundamentally change the interpretation of the results.

Response: We thank the reviewer for raising this important concern. We agree it is essential to define whether the cells are within phagocytes or not. In order to investigate this, we examined phagocyte recruitment to small (1-4 cryptococcal cells) and larger (>4 cells) cryptococcal masses within inter-segmental vessels at 3dpi when dissemination events are most likely. These data have been added (Fig. 6 L-M), with illustrative images of no phagocyte recruitment, both macrophage and neutrophil recruitment, and a macrophage only association (Supplementary Fig. 5). We find that the majority (~70%) of cryptococcal masses have no association with phagocytes, demonstrating that the vast majority of cryptococcal masses are extracellular or with very little immune cell involvement. In addition, the clodronate data (response above) supports the conclusion that the dissemination events we observe do not require Trojan horse macrophages.

In the text as well as the figure legends for figure 6D, G, and H, the authors say that they are labeling VE-cadherin itself to investigate the junctions within the endothelial cells. However, this is completely unsupported by the genetic scheme the authors use wherein a VE-cadherin driven Gal4FF module is used to drive a UAS:Teal cassette that is in no way directly fused to VE-cadherin. Further experiments with a directly linked VE-cadherin would be needed to establish this phenomenon. Also, in the legend, the authors repeatedly refer to VE-cadherin tight junctions but VE-cadherin is only directly recruited into adherens junctions and determining tight junction formation within the vasculature would require different reporters.

Response: We thank the reviewer for this correction and have amended the erroneous reference to “tight-junctions” throughout, referring to these as vascular endothelial cells/junctions. The line was previously published by our co-author (Lagendijk, Nature Communications, 2017) and works as described by the reviewer. We did not intend to claim to label VE-cadherin itself; we have amended text to ensure this is clear (lines 94 and 307).

The authors repeatedly refer to vasculature rupture, but it is unclear what their criteria is for this event. As vascular rupture is a central phenotype to their findings, the authors should further prove that vascular rupture is occurring by showing either leakage of blood cells from the vessel in timelapses or, if blood flow is blocked by cryptococcoma, they could show substantial leakage of larger fluorescent dextrans from the ruptured vessel.

Response: We thank the reviewer for this comment and agree this is an important concern. To address this, we completed experiments using fluorescent dextran injected into the blood stream at 5dpf following *C. neoformans* infection at 2dpf. We have added data to Fig 6 (Fig. 6F and G) showing that only a limited amount of dextran is observed in blocked blood vessels, in comparison to un-blocked vessels, and we suggest this is likely due to loss of blood flow. However, as sites of major *C. neoformans* dissemination, dextran is observed in the surrounding somite tissue indicating vessel rupture.

Please also see response to **reviewer #1 comment 2** about the limitation of dextran with regard to blocked vessels limiting dextran flow to sites of blockages and the addition of data to support the leakage of dextran at sites of extensive dissemination (starting line 296).

The authors use an ADAM10 inhibitor to increase vascular tension to demonstrate that this enhances vascular rupture and Cryptococcus dissemination. However, ADAM10 is a pleotropic molecule, having many effects on vascular and other cells through direct cleavage of proteins involved in cell-cell junctions, cytokine and growth factor signalling, neurotransmission and other pathways. It is difficult to conclude that the effects of the ADAM10 inhibitor are solely due to its effects on vascular tension. The authors should consider using an alternative genetic or chemical genetic approach to increase vascular tension and see if they observe similar phenomena. Alternatively, the authors could try to decrease vascular tension (e.g. ROCK inhibitors) and see if this reduces Cryptococcus dissemination. It would also boost this finding to additionally characterize the rate of vascular rupture during Cryptococcus infection in these small molecule treatments.

Response: We thank the reviewer for this suggestion. With regard to the reasons for use, and interpretation of the ADAM10 data, we have amended our wording for clarity that vascular tension is just an effect this treatment may have (also see **response to #2 minor comment 6).**

We agreed an alternative approach would be beneficial and have attempted this. We identified and tested a ROCK inhibitor (y-27632) at a concentration of 45um, which has been demonstrated to reduce vascular tension in zebrafish but does not cause a fully inhibitory effect (Lagendijk, Nature Communications, 2017). This concentration was used because higher concentrations cause developmental defects in larvae. Our results (below) show a possible trend of reduced cryptococcal dissemination, but this is not significant. This is likely due to the requirement of using low concentrations which do not interfere with larval development, but which are known not to work

100% efficiently. We cannot increase the y-27632 treatment concentration due to likely larval developmental defects. These data have not been included in our revisions for these reasons.

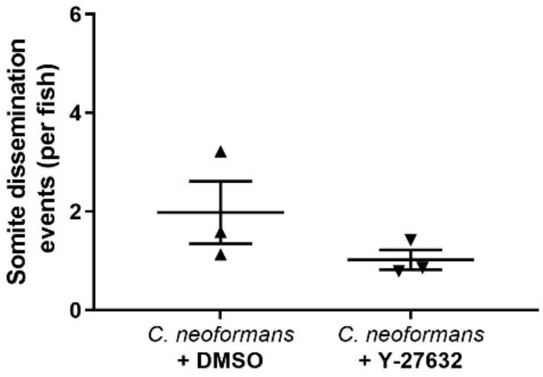

Part III – Minor Issues: Editorial and Data Presentation Modifications

Reviewer #1

Abstract line 38 “stiffness”

Response: Reworded to “tension”.

Figure 1D and text "sufficient to prevent passage of blood cells in blocked vessels". Image does not clearly show blockage by mass (indicated by white arrowhead - missing in legend) of blood vessel. I have no doubt that this occurs but could the authors quickly generate some images or movies from a gata1 transgenic animal or a better contrasted and framed version of current panel?

Response: See response to **reviewer #2 major issues comment** where we have added the requested data (Fig 1E) and added the arrowheads in the legend.

Line 119: I am not an expert in the terminology, but could the intracellular "cryptococcal masses" be called microcolonies?

Response: Reworded throughout to “cryptococcal mass(es)” which we believe best describes the cryptococcal cells and capsule which form the masses.

Line 122: cryptococcal = cryptococci

Response: Corrected.

Regarding the use of mCherry and GFP Cn starting in Figure 2: Please include comment or data demonstrating equivalent growth and virulence between the GFP and mCherry-labelled strains.

Response: As above, (response to **reviewer 2 minor comment 2**). We have added a sentence and citation that demonstrate that the two fluorescent lines have corresponding growth and infection dynamics (line 137).

Figure 2B: Do the larvae overwhelmed by infection from the missing 48.4% have daily imaging data available? Would it be acceptable to presume their outcome at the last alive

time point? I believe their inclusion would greatly increase the statistical power of this experiment.

> **Response:** We thank the reviewer for raising this point and apologise for the lack of clarity. It is not possible to include these larvae because the infection was not the cause of exclusion, as the larvae never reached an overwhelming infection, we cannot include these in our data set. The sentence (line 149) has been amended as it was incorrect to state that some of these 48.8% had an overwhelming infection. Rather the causes of exclusion were, death related due to difficult consecutive re-mounting for imaging, or due to the low infective dose, the infection was efficiently controlled.

Line 154: I am not convinced that the data in Figure 2 links intravascular cryptococcal masses to clonal expansion. It is clear, at this stage of the manuscript, that masses are predominantly seeded by a single cell. I suggest rewriting this summary statement to not overreach.

> **Response:** We have rewritten this summary sentence to ensure we conclude what is shown at this stage of the paper.

Line 155: use of the word “cryptococcoma”, are these masses truly cryptococcomas? Authors have demonstrated a lot of capsule production by the intravascular aggregates suggesting extracellular location. The zebrafish live imaging literature tends to show these capsule-producing intravascular yeasts are not within macrophages (Tenor 2015 for example).

> **Response:** Reworded to “cryptococcal mass” as above.

Line 157: Please stick to "cryptococcal mass" or "cryptococcoma" throughout manuscript. I read cryptococcal mass to mean intravascular cryptococcal mass, is this correct?

> **Response:** Reworded throughout to “cryptococcal mass” to describe the intravascular cryptococcal mass.

Fig 3A. I have to say that this is a quantification that I do not see often.
Consider relabelling the Y-axis "Days post infection that event is observed" or similar.
Can the individual subjects be demonstrated by matching lines? It could get messy but will reinforce the point.
Is there a way of presenting this with time on the X axis?

> **Response:** We have altered the y-axis to “Days post infection that event is observed” and have tested the matching lines (graph on right vs current on left). We believe the left graph is the clearer of the two, but will defer to the editor on this decision.

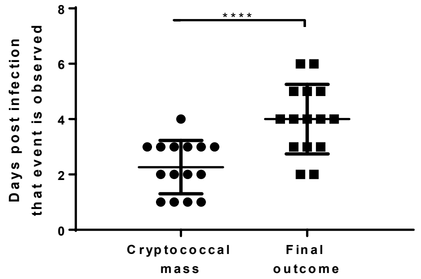

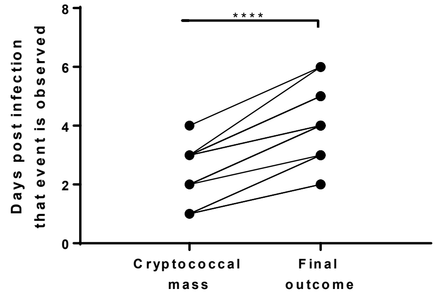

Fig 3B. Is this a separate set of animals from the data in 3A? The day of final outcome does not match and I count 13 data points which does not match any of the individual biological replicates.
When were cryptococcomas observed for this panel? Can additional data points from 3A be used to increase the power of the analysis in 3B?
Does "non-linear regression, one-phase decay" provide statistical testing of the correlation between number of masses and infection progression?

> **Response:** For Fig 3B, the same set of animals are used in data of Fig 3A. The reason that there are 13 vs 15 observed data points is that 3 animals have the same data point, as demonstrated in the graph below. All animals were included in the analysis of both Fig 3A and Fig 3B.
>
> 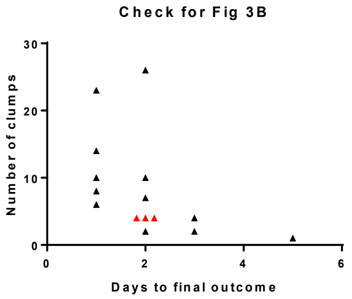
>
>
> The other key difference is that Fig 3A is the initial cryptococcal mass observed to time of final outcome, whereas Fig 3B is the maximum number of cryptococcal masses against the time of final outcome. To ensure this is clearer for Fig 3B, we have amended the results sentence (line 167) and the figure legend.
>
> The non-linear regression, a one-phase decay trend-line was used to demonstrate the trend only. It is standard practice to create a trend line which best fits the data to demonstrate the trend only - as we have done. Although the statistical software can provide an R squared value, this metric is not considered valid for non-linear regression (Spiess and Neumeyer, 2010).

Fig 4A and B. Which vessels were targeted for analysis? Large or small?
Please format 4A like 4B for visual clarity, currently there is no obvious difference in vessel width between the columns in 4A. If you want to use the same Y axes, can you plot as a single panel?

Response: We thank the reviewer for this comment and have made amendments for clarity. The vessels used for analysis in these graphs were the narrow inter-segmental vessels. The legend sentences for Fig 4A and 4B have been altered to "intersegmental vessels" and Fig 4A has been formatted like 4B.

Line 205: rewrite title

Response: Reworded to "Cryptococcal cell size affects the frequency of blood vessel occlusion"

Lines 233-234: it looks like vessels are wider when a mutant mass is present from the associated graph.

Response: This is correct, and we have amended our interpretation and report of the data in Fig 5D and E (lines 267) to include this observation.

Lines 235-237: there is no data on the size of masses to justify this statement.

Response: With the above comment, we have amended the wording to comment on trapping and vessel size only.

Fig 6G and H: Panels are mixed up. Consider moving H up the order to demonstrate normal VE-cad distribution.

Response: We have made swaps to the order of Fig 6 to ensure that the Figure 6 figure order is the same as mentioned in the text.

Lines 270-271: "a clear decrease in VE-cadherin expression" needs more explanation about what the CFP and YFP components measure

Response: Apologies for the lack of clarity of this statement. We have now added the following explanation restating the functionality of the previously published zebrafish VE-Cadherin tension sensor:

As previously described (24), the ratio of the FRET excitation of YFP (FRET signal) intensity to the intensity of independent direct laser excitation of YFP is a measure of the distance between VE-cadherin adhesion proteins and therefore an indirect measure of the vessel tension (i.e. increased tension results in a decrease in FRET signal due to an increase in distance between VE-cadherin molecules). Measuring the FRET/YFP ratio we found a clear decrease in FRET signal between infected and uninfected zebrafish, indicating an increased tension in the vessels during cryptococcal infection (Fig. 7A-C).

In addition, we have added the following to the figure 7 legend:

A-B FRET=YFP signal via FRET with excitation of CFP, CFP=CFP signal with direct excitation of CFP, YFP=YFP signal with direct excitation of YFP**.**

Line 306: This is the first mention of increased blood flow. Measure blood flow by live imaging to validate hypothesis or remove speculation.

Response: As above (reviewer 2 major comment), the impact of blood flow is now mentioned twice in the results section (line 116 and 296) with the evidence supporting our discussions.

Line 345: “observed in during” delete one

Response: “during” has been removed.

Line 444 "Volocity software was used", for what?

Response: Amended “Volocity software was used with this microscope for image capture”

Line 445 "flourecense" typo

Response: corrected

Reviewer #2: see above

Reviewer #3: The authors should substantially rewrite the section population bottlenecks for clarity (line 124-156)

Response: We have made several alterations to the text in this section to improve clarity.

In figure 5 and figure S2-S4 the authors present data as %blocked vessels. How many vessels were analyzed and which blood vessels were analyzed?

Response: We have amended the figure legends to read “% of all inter-segmental vessels” for Fig 5, and S2, S3 and S4.

Figure 6 panel A and AI - As the legend notes that these larvae were serially imaged, it would be useful to show the longitudinal images showing that the Cryptococcus disseminated out of the vasculature. As there are many Cryptococcus in the area, it would be helpful to indicate the cells the authors consider disseminated and would also be helpful to include a phase contrast image or vascular marker to show the localization of the disseminated cell to the somite.

Response: We have added an arrow to Fig 6AI to highlight the clear somite disseminated. We already show a better example of cryptococcal dissemination over time in Fig 1C.

In figure 6B the authors present data that the disseminating Cryptococcus is the same colour as the cryptococcoma in the adjacent vasculature. However, it is really unclear exactly what this graph is showing us, the authors should consider an alternative presentation approach. Also, the raw number of events is uninformative if we do not know how many total events occurred.

Response: We apologise for the lack of clarity. The number of total events (19) is mentioned in the text (line 287), the sentence just before mention of Fig 6B; we have now added this to the figure legend as well. As such, the graph demonstrates that 100% of cryptococcal dissemination events are of the same strain colour as the closest cryptococcal mass, with the graph allowing statistical analysis on the source of GFP and mCherry strains at sites of dissemination.

In figure 6 D, G and H, the authors refer to GFP VE-cadherin but none of the VE-cadherin transgenes they mention in the text is GFP labelled, what transgene is this?

Response: This transgenic is mentioned in the text "**"***Tg*(*10xUAS:Teal*)*uq13bh* (Lagendijk *et al.*, 2017) crossed to endothelial *TgBAC*(*ve-cad:GALFF*)(Bussmann and Schulte-Merker, 2011) for stable expression" which results in a teal coloured fluorescence in VE-cadherin expressing cells. The GFP mentioned in the legend was a reference to the colour used in the figure which has been removed for clarity.

The authors mention in their experimental section that they took 50 μm z-stacks for their VE-cadherin data. However, for the images in the figures, how is the data presented? Is it a single plane or maximum projection of the z-stack? This should be noted in the legend. In particular, this is an important consideration in vascular integrity as single images may not capture the whole of the vasculature.

Response: The images of the cadherin data, and KDRL data (Fig 6 C – J), are single slices, 5μm slices of a 50μm z-stack section; this information has been added to the legend. Before slices were chosen the entire stack was analysed and as such each image is representative of each individual vessel.

Overall, this manuscript could benefit from substantial rephrasing for clarity as well as copy editing.

Response: We have made many amendments throughout the manuscript for improved clarity.

Reference list

Bussmann, J. and Schulte-Merker, S. (2011) 'Rapid BAC selection for tol2-mediated transgenesis in zebrafish', *Development*, 138(19), pp. 4327–4332. doi: 10.1242/dev.068080.

Chen, S. C. A. *et al.* (1997) 'Phospholipase Activity in Cryptococcus neoformans: A New Virulence Factor?', *Journal of Infectious Diseases*. Oxford University Press, 175(2), pp. 414–420. doi: 10.1093/infdis/175.2.414.

Lagendijk, A. K. *et al.* (2017) 'Live imaging molecular changes in junctional tension upon VE-cadherin in zebrafish', *Nature communications*, 8(1402). doi: 10.1038/s41467-017-01325-6.

Santangelo, R. *et al.* (2004) 'Role of Extracellular Phospholipases and Mononuclear

Phagocytes in Dissemination of Cryptococcosis in A Murine Model', *Infection and Immunity*. Infect Immun, 72(4), pp. 2229–2239. doi: 10.1128/IAI.72.4.2229-2239.2004.

Lo Sardo, V. *et al.* (2012) 'An evolutionary recent neuroepithelial cell adhesion function of huntingtin implicates ADAM10-Ncadherin', *Nature Neuroscience 2012 15:5*. Nature Publishing Group, 15(5), pp. 713–721. doi: 10.1038/nn.3080.

Spiess, A. N. and Neumeyer, N. (2010) 'An evaluation of R2as an inadequate measure for nonlinear models in pharmacological and biochemical research: A Monte Carlo approach', *BMC Pharmacology*. BioMed Central, 10(1), p. 6. doi: 10.1186/1471-2210-10-6.
